# Molecular Basis of Inhibitory Activities of Berberine against Pathogenic Enzymes in Alzheimer's Disease

**DOI:** 10.1100/2012/823201

**Published:** 2012-01-04

**Authors:** Hong-Fang Ji, Liang Shen

**Affiliations:** Shandong Provincial Research Center for Bioinformatic Engineering and Technique, Shandong University of Technology, Zibo 255049, China

## Abstract

The natural isoquinoline alkaloid berberine possesses potential to treat Alzheimer's disease (AD) by targeting multiple pathogenic factors. In the present study, docking simulations were performed to gain deeper insights into the molecular basis of berberine's inhibitory effects against the important pathogenic enzymes of AD, that is, acetylcholinesterase, butyrylcholinesterase, and two isoforms of monoamine oxidase. It was found that the theoretical binding affinities of berberine to the four enzymes are very close to the experimental values, which verify the methodology. Further inspection to the binding modes found that hydrophobic interactions between the hydrophobic surface of berberine and neighboring hydrophobic residues are the principal forces contributing to the ligand-receptor interactions. Although berberine cation also has potential to form electrostatic interaction with neighboring residues, it is interesting to find that electrostatic force is excluded in the four cases unexpectedly. These results have important implications for the berberine-based anti-AD drug design.

## 1. Introduction 

**Figure 1:**
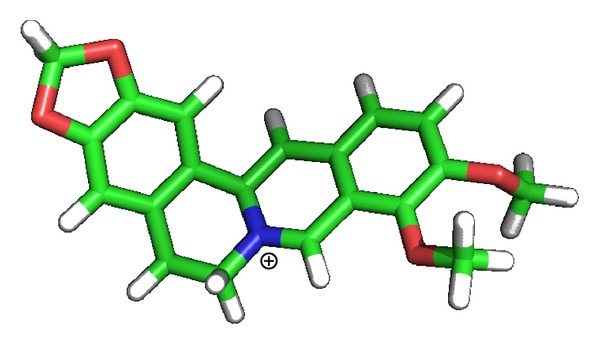
Chemical structure of berberine.

As a natural isoquinoline alkaloid isolated from the Chinese herb Rhizoma coptidis, berberine (Figure 1) has gained considerable attention because of its wide spectrum of biochemical and pharmacological potentials, including antioxidant, antiinflammatory, anticancer activities, and so forth, [1–6]. Alzheimer's disease (AD) is the most common form of degenerative dementia with an estimated prevalence of 30 million people worldwide, and with the accelerated aging of human society, its prevalence is expected to rise steadily [7–10]. In recent years, multiple lines of evidence support that berberine also possesses potential to act as a multipotent agent to treat AD [11–14]. For instance, many experimental studies reported that berberine exhibits inhibitory effects against several key enzymes implicated in the pathogenesis of AD, including acetylcholinesterase (AChE), butyrylcholinesterase (BChE), and monoamine oxidase (MAO) [14–22]. With the aim to elucidate the molecular basis of berberine's inhibitory effects against the pathogenic enzymes in AD, in the present study, the binding modes of berberine with four enzymes, that is, AChE, BChE, MAO-A, and MAO-B, were investigated by means of docking simulations. The results indicate that hydrophobic interactions are the principal forces contributing to the binding of berberine to the four enzymes. Despite the cation ion in berberine structure (Figure 1) can interact readily with the negatively charged acidic residues, no electrostatic force is observed unexpectedly in the four cases. The findings have important implications for the berberine-based anti-AD drug design.

## 2. Methods

### 2.1. Structural Models

Structure coordinates for AChE, BChE, MAO-A, and MAO-B were taken from the Protein Data Bank (PDB codes: 1EA5 [23], 1P0I [24], 1O5 W [25], and 1GOS [26], resp.). The 3D structure of berberine was firstly constructed using standard geometric parameters of SYBYL software and then was optimized using Powell method with the Tripos force field (distance-dependent dielectric) to reach a final energy convergence gradient value of 0.001 kcal/mol.

### 2.2. Docking Methods

The Surflex-Dock program interfaced with SYBYL software is employed to perform docking experiments in this study, which uses an empirically derived scoring function based on the binding affinities of protein-ligand complexes and on their X-ray structures [27]. As a flexible docking method, Surflex-Dock has been proven to be efficient in treating numerous protein receptors [27, 28]. The active sites for four targets were selected on the basis of experimentally reported key residues, which play key roles in their catalytic activities [23–26]. During the simulations, the Kollman-all atom charges were assigned to protein atoms using SYBYL software. For berberine molecule, 30 conformations were selected to dock with target in each run. Standard parameters were used to estimate the binding affinity characterized by Surflex-Dock scores. Surflex-Dock scores (total scores) are expressed in −log10(*K*_*d*_) units to represent binding affinities [29, 30].

## 3. Results and Discussion

The theoretical binding constants of berberine to AChE, BChE, MAO-A, and MAO-B are estimated and listed in Table 1. It can be seen that berberine possesses inhibitory activity against the four enzymes and the respective binding affinities vary largely. The theoretical *K*_*d*_ of berberine to AChE (0.66 *μ*M), BChE (3.31 *μ*M), MAO-A (105.2 *μ*M), and MAO-B (66.0 *μ*M) are very close to the experimental values (Table 1), which verify the accuracy of the present methodology. According to the theoretical *K*_*d*_, the inhibitory activity of berberine against AChE is the highest among the four enzymes, which is in agreement with the experimental results.

**Figure 2:**
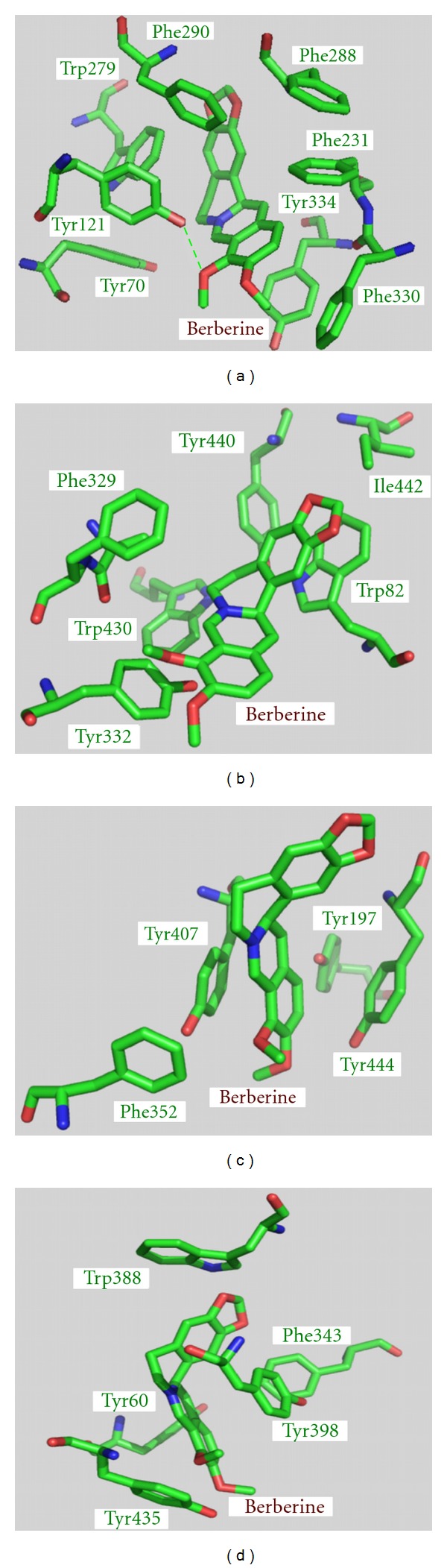
Close-up views of binding modes of berberine in AChE (a), BChE (b), MAO-A (c), and MAO-B (d). The hydrogen bond is marked in green dotted lines.

To elucidate the forces contributing to the binding affinity, the binding modes of berberine in AChE, BChE, MAO-A, and MAO-B are shown in Figure 2. From the molecular structure point of view, berberine has a large hydrophobic surface and a cation ion, which is ideal for interacting with the hydrophobic residues and the negatively charged acidic residues (Figure 1). As shown in Figure 2, the neighboring residues to berberine in the four enzymes are almost all aromatic and/or hydrophobic amino acids. Therefore, these residues can readily form hydrophobic interactions with the hydrophobic surface of berberine. According to Figure 2, there are eight hydrophobic residues (four phenylalanine, three tyrosine, and one tryptophan) interacting with berberine in AChE, while only six hydrophobic residues (one phenylalanine, two tyrosine, two tryptophan, and one isoleucine) with respect to the binding pocket in BChE. Also, a hydrogen bond is formed between berberine and Tyr121 in AChE (Figure 2), which will strengthen the binding affinity and enhance the inhibitory activity of berberine against AChE. These two aspects may account for the relatively stronger binding of berberine to AChE than BChE. In addition, there are less hydrophobic residues involved in the binding of berberine to MAO-A and MAO-B (Figure 2), which results in their much lower binding affinity.

Although berberine cation also has the potential to form electrostatic interaction with neighboring residues in four enzymes, it is interesting to find that as no corresponding negatively charged acidic residues exist at proper positions, no electrostatic interaction is observed. Therefore, according to the present results, the inhibitory activities of berberine against four targets mainly arise from hydrophobic interactions.

## 4. Conclusions

In conclusion, the theoretically estimated binding affinities of berberine to the four enzymes, AChE, BChE, MAO-A, and MAO-B, are very close to the experimental values. According to the binding modes, the hydrophobic interactions between berberine and surrounding hydrophobic residues in the enzymes play predominant roles, while electrostatic force is excluded in the binding of berberine to the four targets. These findings shed lights on the molecular basis of the inhibitory effects of berberine against the enzymes implicated in the pathogenesis of AD and will be helpful for the berberine-based anti-AD drug design.

## Figures and Tables

**Table 1:** Theoretically estimated binding constants (*K*_*d*_) of berberine with AChE, BChE, MAO-A, and MAO-B, and experimental IC_50_.

Targets	Theoretical *K*_*d*_ (*μ*M)	Experimental IC_50_ (*μ*M)
AChE	0.66	0.44 [14], 0.58 [15], 0.37 [16]
BChE	3.31	3.44 [14]
MAO-A	105.2	126 [19]
MAO-B	66.0	98.2 [20], 98.4 [21]
